# Duodenal perforation nine months after accidental foreign body ingestion, a case report

**DOI:** 10.1186/s12893-019-0594-5

**Published:** 2019-09-10

**Authors:** Chi Li, Chee-Chien Yong, Domelle Dave Encarnacion

**Affiliations:** Department of Surgery, Kaohsiung Chang Gung Memorial Hospital, No.123, Dapi Rd., Niaosong Dist., Kaohsiung City, 833 Taiwan, Republic of China

**Keywords:** Foreign body, Duodenum, Perforation

## Abstract

**Background:**

Foreign body ingestion is a scenario occasionally encountered in the emergency room. Pediatric and psychiatric patients are the two most common populations suffering from accidental or in some cases intentional ingestion of foreign bodies. Commonly, majority of cases require no specific treatment and the swallowed objects pass through the digestive tract spontaneously without causing any significant complications. Less than 1% of the cases complicates with gastrointestinal tract perforation, which are often caused by sharp objects, which warrants surgical intervention. The average time from foreign body ingestion to development of perforation was noted at 10.4 days in previous reports. These cases often present in rapidly progressing peritonitis and are subsequently managed by emergent laparotomy. In this case report, we describe an accidental chopstick ingestion of a patient who initially was misdiagnosed and remained asymptomatic for nine months, then presented with acute abdomen.

**Case presentation:**

A 27-year-old man accidentally ingested a wooden chopstick and sought consult at a clinic. Negative abdominal plain film misled the physician to believe ingested chopstick was digested into fragments and passed out unnoticed. The patient presented acute abdomen caused by duodenal perforation nine months later and was subsequently treated with emergency laparotomy with primary duodenorrhaphy.

**Conclusions:**

Negative plain films are not sufficient to conclude a conservative treatment in foreign body ingestion. Computed tomography scan or endoscopic examinations should be done to rule out retained foreign body within gastrointestinal tract.

## Background

Ingestion of foreign bodies may be encountered from time to time in clinical practice. Small objects like coins, buttons or toy compartments are frequently accidentally swallowed by children whereas particular objects are associated with intentional ingestion by psychiatric patients. In prior studies, around 1% of foreign body ingestion complicates with significant clinical sequela like gastrointestinal (GI) tract obstruction or hollow organ perforation [1, 2]. These conditions are often caused by large or sharp objects with subsequent development of unpleasant symptoms shortly after the accidental ingestion which will prompt patients to seek medical consult.

To the best of our knowledge, few reports have described upper GI tract perforation associated with foreign body ingestion which developed more than half year from the time of ingestion to onset of symptoms [3]. We intend to present a case of an accidental ingestion of a chopstick (11 cm in length), of which the perforation of duodenum occurred nine months after the accidental ingestion.

## Case presentation

A 27-year-old young man sought consult at the outpatient clinic of our hospital complaining of sudden onset of dull abdominal pain over the right upper quadrant (RUQ) right of 3-days duration. He denied nausea, vomiting, diarrhea, or any other gastrointestinal symptoms. Prior to his consult at our hospital, he initially sought consult at a local clinic on the second day of symptom onset and was prescribed with antispasmodics under the impression of acute gastroenteritis however there was persistence of the RUQ pain despite the intake of antispasmodics. Persistence as well as progression of the symptoms was noted which now began to radiate to back on the third day which prompted the patient to seek consult at our institution (Table 1).

**Table 1:** Timeline of history, intervention and outcomes

History and treatment course				
Date	Symptoms/signs	Events	Interventions	Outcome
July 2017	Asymptomatic	Local clinic visit	Abdominal plain film: negative Observation	Remained asymptomatic
April 17, 2018	RUQ abdominal pain	Local clinic visit	Antispasmodics	Persisted abdominal pain
April 19, 2018	RUQ abdominal pain with radiation to back	Outpatient clinic visit	Abdominal CT: foreign body penetration over duodenum	Admission Antibiotic treatment
April 20, 2018	RUQ abdominal pain with radiation to back		Laparotomy Remove foreign body Duodenorrhaphy	
April 26, 2018	Complete resolution of abdominal pain	On soft diet		
April 28, 2018				Discharge

The patient had stable vital signs at our outpatient clinic. He denied any chronic illness or surgical history. He denied psychological disorder as well, and drank only in social occasions. The patient however volunteered the history of the accidental ingestion of a wooden chopstick nine months prior to which he sought consult a few days after that incident. The attending physician requested for an abdominal plain film which turned out to be unremarkable and offered the explanation to the patient that the chopstick may have been digested into fragments and will pass out unnoticed. After that consult, patient remained asymptomatic for nine months.

**Fig. 1:**
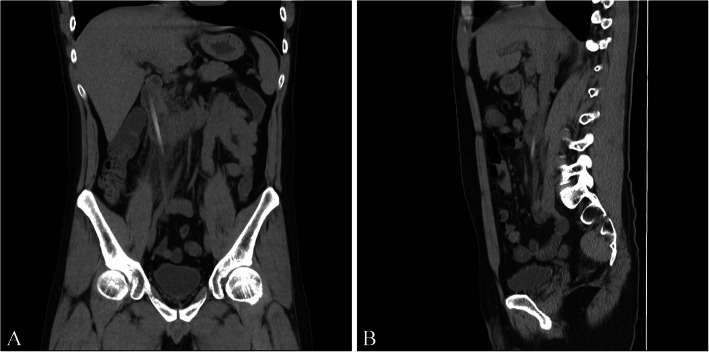
Abdominal computed tomography at the time presentation. A long, radiopaque foreign object was found in the second portion of duodenum piercing into retroperitoneal cavity. **a**) Coronal view **b**) Sagittal view

Upon examination, he presented with a mild abdominal tenderness over the RUQ with negative peritoneal sign. His chief complaint was the back pain with positive knocking pain was noted over the T12 to L2 level. Laboratory investigation revealed leukocytosis (white blood cell count: 12,100 cells/μl) and elevated C-reactive protein (CRP) level (serum CRP: 164 mg/dl). Abdominal computed tomography (CT) was done, which revealed a chopstick-shaped foreign body about 11 cm in length penetrating the second portion of duodenum into retroperitoneal space. Focal fat stranding and tissue swelling were likewise noted but no free air or ascites (Fig. 1).

**Fig. 2:**
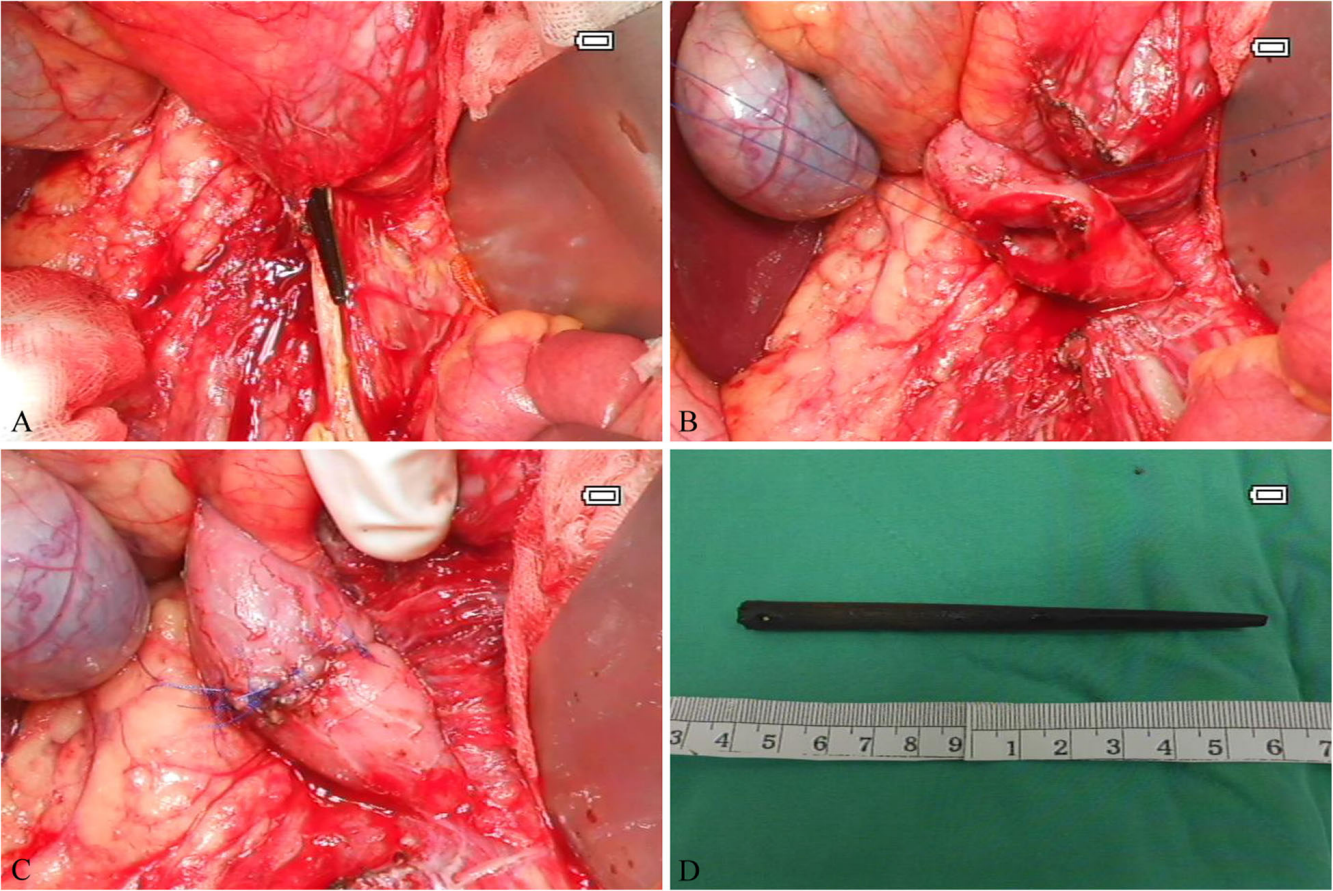
Operative findings. **a**) Chopstick identified by approaching second portion of duodenum via Cattell-Braasch maneuver **b**) Debridement was done to remove inflammatory tissue **c**) Primary duodenorrhaphy with Prolene 3–0 suture **d**) The 11 cm swallowed chopstick

Patient was admitted and started on a broad spectrum antibiotic Ertapenem and was scheduled for emergency exploratory laparotomy on the fourth day of symptom onset. Retroperitoneal space was approached by Cattell-Braasch maneuver which revealed a one piece of chopstick piercing the junction of second and third potion of duodenum at anti-mesenteric side. Minimal amount of abscess and tissue debris was noted along the tract of chopstick (Fig. 2). Careful removal of the chopstick was done and closure of the 4 mm perforation with primary duodenorrhaphy after appropriate debridement. Two open drains were placed for monitoring of leakage before abdominal closure. Minimal blood loss (less than 20 ml) was recorded.

The patient recovered well after surgery. Sips of water were started on the 2nd post-operative day and soft diet on the 6th post-operative day. There were no note of febrile episode or recurrence of abdominal or back pain after surgery. Patient was discharged stable on the 8th post-operative day.

## Discussion and conclusions

Foreign body ingestion in adults are often related to mental retardation, alcohol drinking, or psychiatric disorder [4, 5]. The case mentioned in this report was mentally normal, although at the time of the accidental ingestion, the patient was alcohol intoxicated. In previous reports, small objects generally pass through GI tract without complications, while large, sharp, or caustic ingested foreign bodies will usually cause an unpleasant symptom or severe complications like GI tract perforation shortly after ingestion [1, 2, 6]. The average time from ingestion of foreign body to occurrence of perforation was 10.4 days [4]. Only one case report from China presented foreign body embedded in esophageal mucosa for ten months before endoscopic removal [3].

Our case was aware of the ingestion of chopstick. The negative clinical symptoms/signs and unremarkable plain film on initial consult misled the initial attending physician, which resulted in delay of proper management. The swallowed chopstick remained intact in his upper GI tract for nine months (from July 2017 to April 2018) before causing significant complication. Such history should alert clinical practitioners that lack of symptoms and negative plain films are not sufficient to conclude a conservative treatment in foreign body ingestions. Computed tomography scan or endoscopic examinations should be done to rule out retained foreign body within GI tract.

Clinical presentation of our case after duodenal perforation was compatible with the statistic results in previous studies [4, 7, 8]. He remained afebrile throughout the entire course, and did not present typical signs of acute abdomen. Instead, his back pain was the chief complaint at the time of consult. Detailed history taking is crucial since patients may not be aware of foreign body ingestion in some cases. The possibility of hollow organ perforation should also be excluded before endoscopic examinations.

## Data Availability

Data sharing is not applicable to this article as no datasets were generated or analyzed during the current study.

## Abbreviations

CRP: C-reactive protein
CT: Computed tomography
GI: Gastrointestinal
RUQ: Right upper quadrant

## References

[1] Velitchkov AG, Grigorov GI, Losanoff JE, Kjossev KT (1996). Ingested foreign bodies of the gastrointestinal tract: retrospective analysis of 542 cases. World J Surg.

[2] Goh BK, Chow PK, Quah HM, Ong HS, Eu KW, Ooi LL (2006). Perforation of the gastrointestinal tract secondary to ingestion of foreign bodies. World J Surg.

[3] Li SX, Li H, Chen T, Xu MD (2014). Endoscopic retrieval of an 18-cm long chopstick embedded for ten months post-automutilation in the esophagus of a patient with psychosis. World J Gastrointest Endosc.

[4] Rodriguez-Hermosa JI, Codina-Cazador A, Sirvent JM, Martin A, Girones J, Garsot E (2008). Surgically treated perforation of the gastrointestinal tract due ingested foreign bodies. Color Dis.

[5] Lai AT, Chow TL, Lee DT, KwoK SP (2003). Risk factors predicting the development of complications after foreign body ingestion. Br J Surg.

[6] Gambardella1C, Allaria1A, Siciliano1 G, Mauriello1 C, Patrone1 R, Avenia N, et al. Recurrent esophageal stricture from previous caustic ingestion treated with 40-year self-dilation: case report and review of literature. BMC Gastroenterol. 2018;18(1):68–72. 10.1186/s12876-018-0801-3 PMC5964928 29788901

[7] Bansod A, Mehsare P, Kolpakwar S, Jantli M, Laxminarayan L (2016). Small bowel perforation secondary to unusual foreign body - a case report. Int Surg J.

[8] Glenera J, Poris S, Foles B, Harmon R (2016). Perforated duodenal diverticulum case report. Int J Surg Case Rep.

